# Analysis of Physiological Responses during Pain Induction

**DOI:** 10.3390/s22239276

**Published:** 2022-11-29

**Authors:** Raquel Sebastião, Ana Bento, Susana Brás

**Affiliations:** 1 IEETA, DETI, LASI, University of Aveiro, 3810-193 Aveiro, Portugal; 2 DFis, University of Aveiro, 3810-193 Aveiro, Portugal

**Keywords:** autonomic nervous system (ANS), cold pressor task (CPT), pain induction, pain assessment, physiological signals, signal processing, signal analysis

## Abstract

Pain is a complex phenomenon that arises from the interaction of multiple neuroanatomic and neurochemical systems with several cognitive and affective processes. Nowadays, the assessment of pain intensity still relies on the use of self-reports. However, recent research has shown a connection between the perception of pain and exacerbated stress response in the Autonomic Nervous System. As a result, there has been an increasing analysis of the use of autonomic reactivity with the objective to assess pain. In the present study, the methods include pre-processing, feature extraction, and feature analysis. For the purpose of understanding and characterizing physiological responses of pain, different physiological signals were, simultaneously, recorded while a pain-inducing protocol was performed. The obtained results, for the electrocardiogram (ECG), showed a statistically significant increase in the heart rate, during the painful period compared to non-painful periods. Additionally, heart rate variability features demonstrated a decrease in the Parasympathetic Nervous System influence. The features from the electromyogram (EMG) showed an increase in power and contraction force of the muscle during the pain induction task. Lastly, the electrodermal activity (EDA) showed an adjustment of the sudomotor activity, implying an increase in the Sympathetic Nervous System activity during the experience of pain.

## 1. Introduction

Pain is a complex biopsychosocial phenomenon caused by damage or potential damage in the tissues and serves a vital protective function. The International Association for the Study of Pain revised the definition of pain as: “an unpleasant sensory and emotional experience associated with, or resembling that associated with, actual or potential tissue damage” [1]. This revision also specified that (a): pain is always a personal experience that is influenced by several factors; (b) pain cannot be inferred solely from activity in the sensory neurons; (c) the concept of pain is learnt throughout an individual life experience; (d) a person’s report of an experience as pain should be respected; (e) pain serves an adaptive role but can also cause effects on individual well-being; and (f) the inability to communicate does not exclude an individual’s capacity to feel pain [1].

In almost every clinical practice, especially in neurological and musculoskeletal problems [2], pain is a common symptom and an accurate assessment is critical to ensure a safe and effective management of pain. Currently, the most used standard method to assess pain is based on self-reports, both in clinical and experimental settings. Despite subjective, self-reports being generally easy to obtain, requiring practically little to no equipment, allowing for comprehensive information collection, and exhibiting typically good reliability [2], these instruments rely on the ability of the individual to process external information and communicate a response, which may not always be feasible. Moreover, the use of self-reports from the patient to assess pain may be hazarded by the age, cognitive condition, and verbal communication capabilities of the patient. In such cases, pain needs to be assessed by a healthcare provider, which is a more complex and time-consuming task, and can be highly challenging due to individual differences in pain expression and behavior and physiological changes not always being specific to pain.

It is thought that pain exacerbates the autonomic response to stress, a rationale supported by evidence showing a neuroanatomical overlap between nociceptive and autonomic pathways [3]. For example, studies have shown that the application of pain stimuli induces significant heart rate acceleration. Therefore, there has been a growing interest in the use of autonomic reactivity as an objective marker of pain, and several studies have investigated physiologic variables for this purpose under pain-induced conditions [2,4,5,6].

The Cold Pressor Task (CPT) is a pain-inducing method that requires individuals to immerse one hand (or forearm) in cold water for as long as they can tolerate it or during a fixed period of time. The main advantages of this method rely on its portability, minimal training to use, and few risks. The primary disadvantage of the CPT is the significant methodological divergences in its implementation and in the measurement of pain outcomes, crippling the comparison of results from different studies [4,5,7,8,9]. There is increasing information linking the feeling of pain with the Autonomic Nervous System (ANS). Therefore, several studies have investigated and recorded the alterations of the ANS with the use of CPT.

### Goals and Organization

As pain is mostly assessed through the use of subjective instruments, such as self-reports relying on a pain-scale, the main goal of the present study was to study and characterize physiological responses when experiencing pain. For that, a pain-inducing protocol was implemented on forty-five healthy-volunteer participants, and several physiological signals (electrocardiogram—ECG; two electromyograms—EMG, electrodermal activity—EDA) were recorded, while, simultaneously, pain was induced through the exposure to cold stimuli. Thus, with this protocol, we analyze the physiological responses of pain and assess the pain perception through self-reports based on a numerical rating scale (NRS). This work is organized as follows: Section 2 presents related works with respect to the ANS reactions associated with induced pain. Section 3 presents the study protocol, data collection, and methodology for data analysis, while Section 4 presents the obtained results. Finally, before presenting the conclusions of our study and identifying further research in Section 6, Section 5 discusses the obtained results regarding the physiological characterization of pain.

## 2. Background

There is increasing information linking the feeling of pain with the ANS. Therefore, several studies have investigated and recorded the alterations of the ANS when participants are subjected to pain-inducing stimuli.

Concerning cold stimuli to induce pain, the work [4] quantifies the changes in skin impedance, Heart Rate (HR), and facial skin temperature when healthy volunteers were subjected to acute pain through a CPT (with water at $0{\;}^{\circ }C$). A total of 19 participants were included in the study. The results showed an increase in all the parameters calculated during the CPT, in comparison to those calculated during the baseline. However, only the skin conductance increase was statistically significant. One possible justification for the minor variation of HR during both conditions (baseline and CPT) may stem from anxiety felt by some participants before the pain-inducing task.

Ref. [5] analyzed the relation between efferent sympathetic nervous system activity to skeletal muscle (MNSA) and pain sensation during localized skin cooling. Ten subjects took part in the study, immersing their right hand in different temperature water baths for three minutes each. The levels of temperature in the bath range from warm ($28{\;}^{\circ }C$ and $21{\;}^{\circ }C$—non-pain inducing) and mid-level ($14{\;}^{\circ }C$) to cold ($7{\;}^{\circ }C$ and $0{\;}^{\circ }C$—pain-inducing). The participants went in order from the warmest to coldest temperature, with a ten-minute interval between the recovery three-minute period of the last water tank and the three-minute baseline of the next. While the study was being performed, the MNSA, Blood Pressure (BP), HR, and breathing were continuously recorded. The observations of this study demonstrated that there was no evident influence on MNSA when the participants were subjected to non-painful skin cooling. During the hand immersion in ice water, there was a progressive rise in MSNA as skin temperature started to decrease. However, there was a more significant peak increase in the MSNA signal during the $0{\;}^{\circ }C$ immersion compared to the $7{\;}^{\circ }C$. Regarding HR, there was a significant rise during the initial phase of the $0{\;}^{\circ }C$, which was expected. Even so, the HR consistently increased in less painful water temperatures, although on a smaller scale. As for the BP, there were no significant changes during the study.

Aiming at studying the relationship between HR and multidimensional aspects of pain (intensity and unpleasantness) in healthy individuals, 39 healthy volunteers were subjected to hot water ($47{\;}^{\circ }C$) hand immersion test for two minutes, while ECG and EDA were being recorded [10]. Participants also had to rate their perceived pain every 15 s using a Visual Analogue Scale (VAS). The HR, Heart Rate Variability (HRV) parameters, and Skin Conductance Level (SCL) were calculated. The study showed a steady rise in pain intensity and unpleasantness, as well as in HR, with the progression of immersion time. These results seemed to indicate a rise in sympathetic activity and a drop in parasympathetic activity, which is in agreement with the usual body reaction to a noxious stimulus. Regarding pain perception and HR, there seems to exist a greater correlation between HR and pain unpleasantness than with pain intensity, suggesting that pain-related autonomic responses are functionally related to the affective dimension of the experience. However, the correlation between pain perception and HR indicated a vast difference between genders, with men presenting much greater values.

In addition, through the use of hot stimulation, Ref. [11] assessed if there was a relation between sudomotor activity and heat pain perception. To that end, a thermal stimulus protocol, using a Peltier type contact thermode, was applied to 22 healthy participants while the EDA was being recorded. The participants also reported their subjective perception of pain through VAS. During the procedure, the baseline temperature of the thermode started at $31.5{\;}^{\circ }C$. Three different types of stimuli were tested on all the participants on three different days. The results indicated a positive correlation between changes in sudomotor activity and pain perception as the mean EDA level and sympathetic skin response were higher in the pain phases. This was especially verified in quicker temperature slopes. Since both features are considered reliable indices of emotion, it is conceivable that the increase in sudomotor activity is also related to an emotional component. After the end of the pain-inducing protocol, the mean EDA decreased, indicating a drop in sympathetic outflow when an event responsible for an emotional response is over.

Ref. [2] studied the alterations in the HR, skin conductance, and VAS ratings in response to noxious stimulus created by calibrated heat stimulus of different intensities, which range from warm to pain-inducing. The data were analyzed from two different perspectives: the correlation between the autonomic response and pain intensity in subjects separately (subject analysis) and the correlation between the average pain intensity and the autonomic responses to the same temperature in all individuals (group analysis). The results demonstrated that an increase in pain intensity generated an increase in both HR and skin conductance. The subject analysis revealed a higher correlation with skin conductance, leading to a belief that this metric is more sensitive to changes in perception. However, the magnitude increases of the skin conductance did not significantly correlate with the magnitude of pain intensity, suggesting that this measure alone does not predict the absolute level of pain reported by the subject. The opposite was true for HR, as it did not reliably predict verbal responses to pain on a subject basis but did on the group level. These differences suggest that, although HR is affected by pain perception, it is a very noisy measure.

With a protocol of several tests to assess the autonomic function and considering patients with chronic neck or shoulder pain and control participants, Ref. [12] studied the differences in responses of muscle blood flow, muscle activity, HRV, and BP. The protocol consisted of an initial 15-min rest period and three different tests with a 5-min rest period in between: the hand grip test (HGT), the cold pressor task (CPT), and the deep breathing test (DBT). During the rest period, patients showed lower parasympathetic activity compared to the control group. Blood flow in the trapezius muscle during HGT and CPT was also lower in patients than in the control group. This result may be the consequence of increased sympathetic activity leading to a change in blood flow due to an imbalance between vasoconstriction and dilation in the affected muscle. Finally, it was observed that trapezius muscle activity in patients was highest during the rest period after static contraction, which seems to indicate an inability on the part of patients to relax properly after static work.

Ref. [13] evaluated the changes in the ANS in patients with fibromyalgia through CPT. A total of 38 women participated in this study, of which 23 were patients with fibromyalgia. At the beginning and end of CPT, the pain was assessed with a numerical scale, and a thermographic recording of the forearm was measured. The physiological measurements considered included BP and pulse rate. It was observed that participants with fibromyalgia had a lower resistance to the stimulus of cold water. These observations may thus be related to the abnormal functioning of the ANS and, therefore, abnormal perception of pain and/or suffering from ischemia more rapidly.

Considering 13 physiological parameters derived from the HR, breath rate (BR), galvanic skin response (GSR), and facial surface electromyogram, the authors of [14] proposed artificial neural network classifiers to distinguish between no, mild, and moderate/severe acute pain. A group of 30 healthy volunteers was subjected to thermal and electrical pain stimulation, and pain was self-reported using VAS. The results show that HR, GSR, and BR were better correlated to pain intensity variations than facial muscle activities. The authors also concluded that the use of multiple physiological parameters for pain classification was revealed to be advantageous, especially in the classification of mild pain category since data from this category overlapped greatly with the other two categories.

Ref. [15] goes beyond the analysis of machine learning recognition models for pain assessment based on physiological and behavioral data. It also proposes a framework for feature extraction methods that allows a fair comparison of the performances of feature extraction and feature learning approaches. The authors concluded that simple feature engineering approaches, relying on features extracted from the signals based on expert knowledge, lead to better performances than deep learning approaches and that more complex deep learning architectures do not necessarily outperform simpler ones. However, although comparing five different approaches evaluated on two databases, the major drawback of this work relies on the use of the EDA signal only. Thus, further research should be endeavored by including other physiological data and by considering data fusion approaches to increase the performance of the pain classification models.

## 3. Materials and Methods

This section describes the protocol for data collection and presents the methods applied for analyzing the body response during the induction of pain through cold pain stimuli implemented as a CPT. The different methods used to analyze the data were implemented in Matlab R2021a (MATLAB R2021a and Simulink R2021a) [16].

### 3.1. Data Collection

Aiming to study the physiological changes that pain provokes, 45 participants were subjected to a pain-inducing protocol (CPT), while, simultaneously, physiological signals, namely ECG, EMG, and EDA, were being collected. This study was approved by the Ethics and Deontological Council of the University of Aveiro (number 09-CED/2019).

All the participants were recruited from the local community, they were healthy, did not suffer from any disease that causes chronic pain, did not present any mental illness or neurological disorder, and, lastly, could comprehend and answer to self-report measures. As explained before, we studied a total of 45 participants, 27 male and 18 female, with ages between 21 and 59 (33 ± 11 years old).

**Figure 1:**
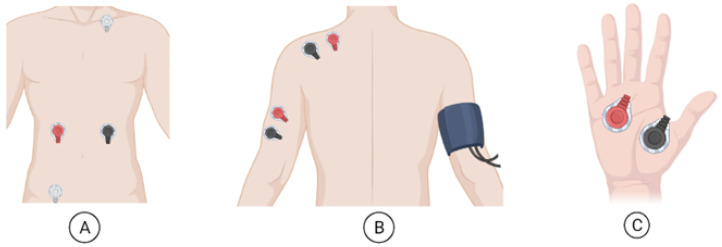
Illustration of the placement sites for the different electrodes (**A**) ECG electrodes plus reference electrode of the EMG; (**B**) EMG electrodes on the trapezius and triceps muscle plus BP monitor; and (**C**) EDA electrodes.

To perform the CPT, two specially designed tanks were used. These were produced to be able to sustain the water at the desired temperature. The physiological data were collected with the Biosignalsplux^®^ Explorer tool kit, with a sampling frequency of 1000 Hz. A total of four sensors were used to record the signals, two EMG sensors, one ECG sensor, and one EDA sensor. The ECG was collected with a triode configuration: two electrodes were placed on the right and left side of the participant’s ribcage, and a reference electrode was placed above the pelvic bone (as shown in Figure 1A). The EMG sensors, with a bipolar configuration, were placed in the trapezius and triceps muscles of the non-dominant arm (as observed in Figure 1B). Since there was no built-in reference electrode, one, serving for both EMG signals, was placed in the clavicle (Figure 1A). The EDA sensor, which also had a bipolar configuration, was collected on the dominant hand (as indicated in Figure 1C). Additionally, to mark the different epochs, a handheld switch directly connected to the hub was used. After collection, the raw ECG, EDA, and EMG signals were converted to microvolts (mV), according to the information provided in the Biosignalsplux sensor datasheets (https://support.pluxbiosignals.com/wp-content/uploads/2021/10/biosignalsplux-Electrocardiography-ECG-Datasheet.pdf; https://support.pluxbiosignals.com/wp-content/uploads/2021/10/biosignalsplux-Electromyography-EMG-Datasheet.pdf; https://support.pluxbiosignals.com/wp-content/uploads/2021/11/Electrodermal_Activity_EDA_Datasheet.pdf). Lastly, the BP was measured at three different moments during the study, with the resource of an upper arm blood pressure monitor that was placed on the bare upper dominant arm of the participant. Participants also had to self-report their level of pain at different moments, using a 0–10 level NRS. With zero score standing for no pain, one to three scores for light pain, four to six scores for moderate pain, seven to nine scores for severe pain, and, finally, a 10 score for the worst pain imaginable.

All the information regarding the study was given to the participants, and the respective informed consent was obtained. At the beginning of the procedure, the participants had to respond to the instrument for data collection regarding their age, gender, and health status, thus ensuring that they complied with the inclusion criteria. That same data collection sheet was later used to fill out their pain level.

The protocol started with a five-minute baseline recording, where the participant had to be seated, at a comfortable position, with their arm close to their body, trying to avoid movements. Afterwards, participants were asked to immerse the non-dominant hand and forearm inside the warm water tank (with temperature $37{\;}^{\circ }C\pm 1{\;}^{\circ }C$) for two minutes, to ensure that all the participants started the CPT with similar skin temperatures. Before the end of this task, the level of pain, with an NRS, was assessed. Afterward, for the induction of pain, the participants immersed the arm into the cold-water tank (with temperature $7{\;}^{\circ }C\pm 1{\;}^{\circ }C$) and the CPT started. If the participant was unable to withstand the CPT for the whole two minutes, they could withdraw their hand from the cold tank. In this case, the participant was advised to notify their wish to remove the arm from the tank and, before doing so, to report their current pain level and the level of the maximum pain experienced during the CPT. If the participant was able to withstand the entire CPT, the current and maximum pain levels were reported at the two-minute mark. Right after removing the arm, the participants reported again the pain level, and the BP was measured. The participant transferred the arm back to the warm water tank for two minutes of immersion. Next, the hand and forearm were dried, and, while seated in a comfortable position, a five-minute rest period, similar to the initial baseline, commenced. At the three-minute point during this rest period (around five minutes after the end of the CPT), they were asked to give their current pain level and to report the maximum level of pain they felt in retrospect.

**Figure 2:**
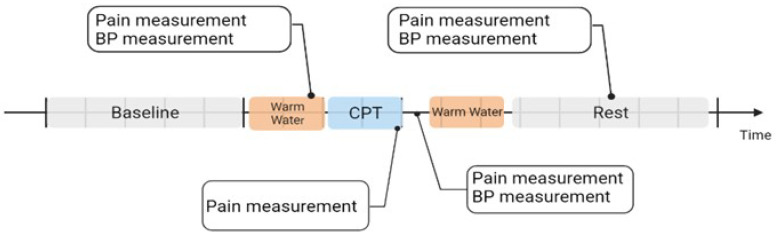
Representation of the different steps of the experimental procedure, with further indication of the moments where pain was self-reported and the BP measured. ECG, EDA, and EMG were continuously recorded throughout the study.

The scheme of the implemented protocol is shown in Figure 2.

### 3.2. Data Analysis

After acquiring the raw ECG, EMG, and EDA signals, they had to be pre-processed. The ECG was filtered considering the frequencies between 0.5 Hz and 40 Hz. The EMG was filtered to remove the interference of the powerline, and high-passed at 10 Hz. Lastly, the EDA signals were low-pass filtered at 10 Hz.

After the pre-processing step, the data were normalized according to the baseline epoch, which corresponds to the first five minutes of this study. The feature extraction was performed using the Neurokit2 (https://neuropsychology.github.io/NeuroKit/) in Python. After the data were processed, it was divided into epochs according to the pressing of the triggers. The five epochs created are the five-minute baseline recording (Baseline), the first two-minute recordings of the hand and arm in the warm water tank (WarmWater1), the CPT recording, the two-minute recordings of the warm water tank for the second time (WarmWater2), and, finally, the last five minute rest (Rest).

Afterward, statistical analysis was performed to investigate differences in the extracted features in several epochs. As all of the features failed to be normally distributed, the differences between the five different epochs were evaluated with the non-parametric Friedmann. When a significant difference was found between the five epochs, the Wilcoxon signed-rank test, with Bonferroni correction, was performed to evaluate which epochs were significantly different from each other.

## 4. Results

Six of the 45 original volunteers had to be taken out of the study, as the participants did not fulfill all the protocol. As such, a total of 39 individuals were used in this study.

The felt pain was assessed through self-report at four moments. On the first pain evaluation, at the end of the WarmWater1, no participant reported pain (NRS = 0). On the second assessment, at the end of the CPT, the average value for the pain of the participants reported using the 0–10 level NRS, at that exact moment, was 6.85 ± 2.23.

After removing the arm from the water, the level of pain decreased around a 1.5 score, with participants reporting a mean of 5.37 ± 2.55. At the final assessment, participants reported their current level of pain and tried to recall their maximum. For the current level of pain, only three participants reported a low level of pain (NRS = 1). As with respect to the recall of the maximum level of pain, participants reported 7.37 ± 2.19. In general, women reported higher levels of pain when compared to men.

### 4.1. ECG Processing and Analysis

Regarding ECG features, the HR was computed and the maximum and minimum values of each ECG cycle were calculated, R peaks and S peaks, respectively. Afterward, for each epoch, the averages of those were computed.

With respect to the HRV features, and due to the different lengths of the epochs and the short term of the CPT epoch, only the following features were considered: RMSSD, pNN50 (time-domain features), and SampEn (nonlinear feature). The description of the used features is presented in Table 1.

**Figure 3:**
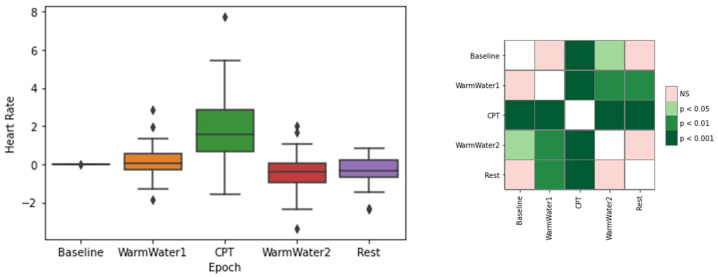
Boxplot of mean HR values for each epoch (**left**) and respective *p*-values between different epochs, with Bonferroni correction (**right**). The ⋄ stands for outliers.

Figure 3 represents the results for the normalized mean HR. It is clear that the most prominent boxplot is the CPT, being the epoch with the higher HR values, showing a response to the stress caused by the pain. Observing the matrix statistical results, there is a statistically significant difference between the mean HR during the CPT and from the remaining epochs. From the obtained results, there appears to be no difference between the Baseline and the WarmWater1, and between the Baseline and the Rest. However, the same was not verified with respect to the Baseline and the second tank of warm water, which may be the reflex of the pain induced during the CPT.

**Figure 4:**
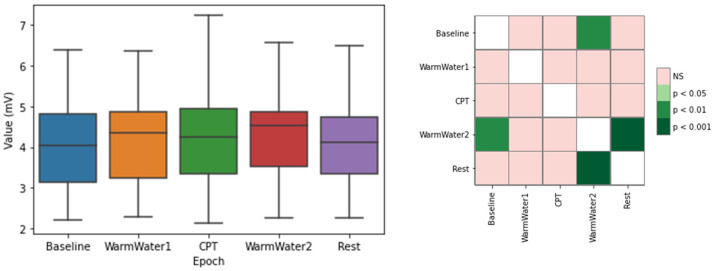
Boxplot for the mean maximum ECG cycle values (**left**) and respective matrix of calculated *p*-values between different epochs, with Bonferroni correction, for the (**right**).

Figure 4 regards the maximum value of the ECG cycles, which correspond to the R-peaks, showing a median value increase for the normalized R-peak amplitude from the Baseline to the WarmWater1, with little variation of the dispersion. This increase is about 7.7% from the Baseline to the WarmWater1. However, this is followed by a decrease of 2.55% during the CPT. The median, rises, once again, reaching its peak with an increase of about 6% during the WarmWater2. The amplitude returns to near its original value during the Rest period. Although slight, there seems to be a reaction when the participants placed their hands on the water. However, there is no significant difference between the non-pain-inducing and pain-inducing water temperatures on the maximum amplitude of the ECG cycles. The statistical analysis corroborates this, as it did not show any inter-epochs significant differences, with the exception of the WarmWater2 for the Baseline and the Rest, the epochs with the highest and lowest amplitude values, respectively. These results suggest that the maximum ECG amplitude is not a suitable feature to examine the presence of pain in an individual when subjected to CPT.

**Figure 5:**
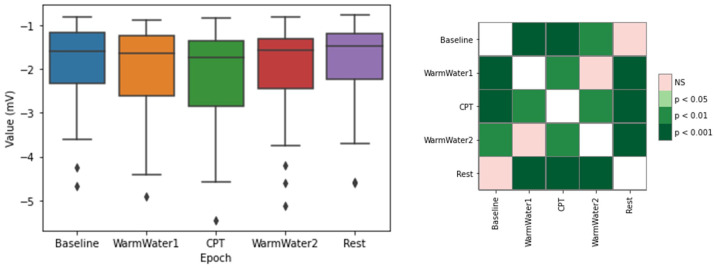
Boxplot for the mean minimum ECG cycles (**left**) and respective matrix of calculated *p*-values between different epochs, with Bonferroni correction, for the (**right**). The ⋄ stands for outliers.

Another ECG feature studied was the minimum value of the ECG cycles, which corresponds to the S-peak. Figure 5 shows a decrease of the median value from the Baseline to the WarmWater1, followed by a decrease from this epoch to the CPT. After the pain-inducing procedure, the minimum amplitude of the ECG cycles gradually increased. The statistical analysis for this feature shows that the CPT had significant differences from all the other epochs, being statistically more significant with the Baseline and Rest periods. There were no significant differences between the Baseline and Rest. Finally, regarding WarmWater1 and WarmWater2, there was, also, no significant difference between them. Nevertheless, both had statistically significant differences from the other groups.

**Figure 6:**
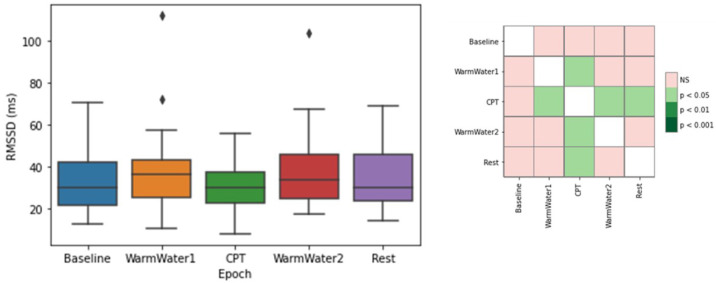
Boxplot of RMSSD values for each epoch (**left**) and respective *p*-values between different epochs, with Bonferroni correction (**right**). The ⋄ stands for outliers.

Figure 6 shows the RMSSD results. Looking at the graph, the epoch with the lowest values is the CPT. As for the other epochs, the RMSSD values are higher. However, the Baseline and, especially, the WarmWater1 appear, in general, to have slightly lower levels when compared to the Rest and WarmWater2 epochs. Finally, analyzing the *p*-values obtained by the Wilcoxon test, there is only a significant difference between the CPT and the WarmWater1 and between the CPT and the following epochs.

**Figure 7:**
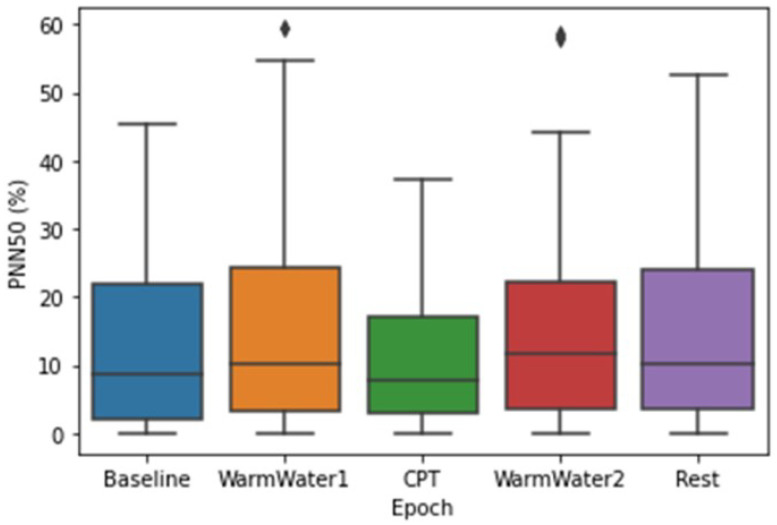
Boxplot of pNN50 values for each epoch. The ⋄ stands for outliers.

Figure 7 displays the pNN50 results. In accordance with the findings of the RMSSD, the epoch with the lowest pNN50 values was the CPT. In this epoch, the participant with the highest pNN50 had less than 40% of their heartbeats longer than 50 ms. Overall, the median values in each epoch seem to be similar. Even so, the epoch with the lowest median was the CPT (7.7%), with a 0.9% difference when compared to the Baseline and 2.4% compared with the WarmWater1 and Rest, while the WarmWater2 was the epoch with the highest median pNN50 (11.4%). There seems to be a consistent positive skewness on the boxplots, which means that the values of the upper quartile are more dispersed. This may be due to natural differences between the participants. Along with the protocol, there is a general increase of values from the Baseline to the WarmWater1, followed by a decrease during the CPT and a subsequent rise during the WarmWater2 and Rest epochs, indicating a recovery after the CPT. Unlike the previous features, there was no significant statistical difference shown between the epochs.

**Figure 8:**
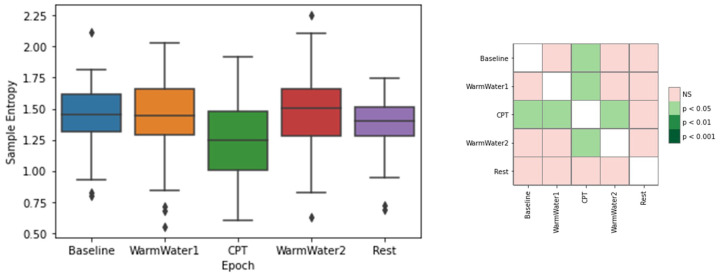
Boxplot of SampEn values for each epoch (**left**) and respective *p*-values between different epochs, with Bonferroni correction (**right**). The ⋄ stands for outliers.

Finally, the regularity and complexity of each epoch are presented in Figure 8, through the SampEn values. The epoch with the lowest value was the CPT. Another interesting observation is the results in the WarmWater2, which had generally higher values and a noticeable increase in the median value, which implies less predictability. Looking at the Baseline and WarmWater1, both have equal median values (1.45). However, the values showed greater dispersion on the latter, which denotes greater behavioral differences in participants when compared to the former epoch. Lastly, the Rest epoch had a similar mean value to the two initial epochs and smaller dispersion, suggesting that, overall, the participants were able to recover after the CPT. The statistical analysis (Figure 8—right) only indicates a statistically significant difference between the CPT and the remaining epochs, with the exception of the Rest.

### 4.2. EMG Processing and Analysis

With respect to the EMG signal, the features described in Table 2 were analyzed.

**Figure 9:**
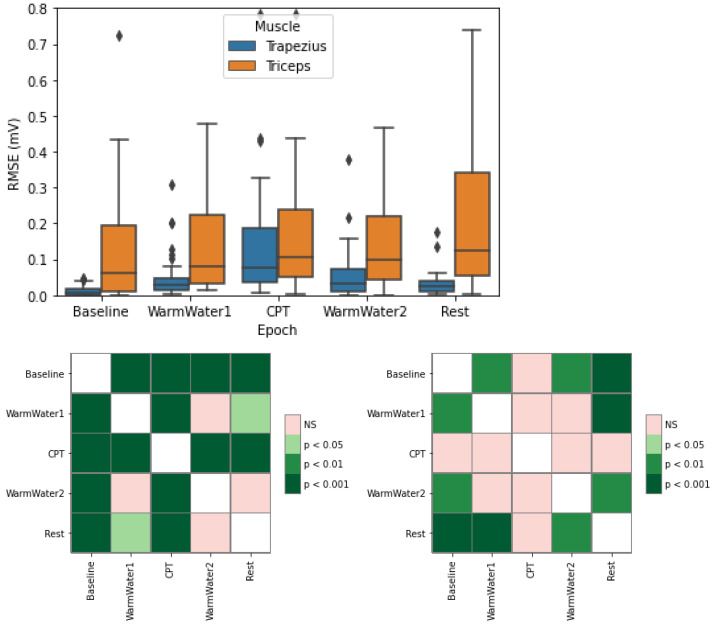
Boxplot of RMSE for the trapezius and triceps muscle (**left**) with respective statistical analysis with *p*-values, with Bonferroni correction, for the trapezius (**bottom left**) and triceps (**bottom right**). The ⋄ stands for outliers.

Figure 9 represents the Root Mean Square (RMS) of the electromyogram (RMSE), which is usually associated with the force a muscle exerts. It is clear that there is a progression in the RMSE values for the trapezius muscle from the Baseline, where the muscle was at rest, until the CPT when the participant has its forearm placed in cold water, experiencing pain, which was then followed by a steady decrease as the participant returned to rest. This suggests that the increase in the not fatiguing muscle contraction during CPT was, presumably, to endure the pain. Focusing on the boxplots, another interesting observation is near to no dispersion of the values during the non-painful epochs indicating a stable behavior from all the participants during these periods. The greater dispersion during the CPT may be due to different individual responses in reaction to pain. Concerning this muscle, the statistical analysis further demonstrates a significantly different behavior during the painful stimuli (CPT) in comparison with all the remaining epochs, especially with the Baseline and Rest epochs. On the contrary, the RMSE values for the triceps muscle did not indicate any type of evolution during the study, except for an increase in the Rest epoch. As for the statistical analysis, there were no significant differences between the values on the CPT and the other epochs, except for the Rest. These results are putting forward that the RMSE is not an adequate feature to study the triceps contraction force, and may be hypothesized that the trapezius is a better muscle to study the effects of pain on the body caused by the CPT.

**Figure 10:**
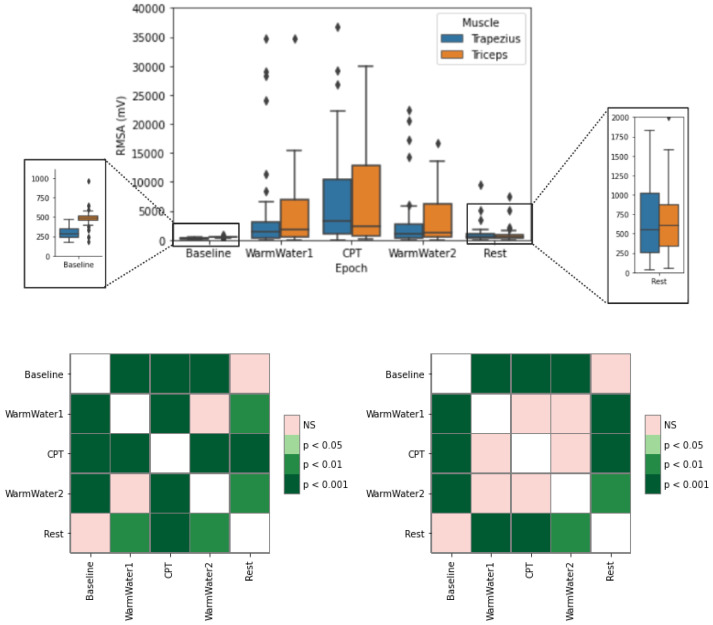
Boxplot of RMSA for the trapezius and triceps muscle (**top**) with respective statistical analysis with *p*-values, with Bonferroni correction, for the trapezius (**bottom left**) and triceps (**bottom right**). The ⋄ stands for outliers.

Figure 10 represents the RMS of the amplitude (RMSA). The results show a general increase in the values from the baseline to the CPT on both muscles. This implies that there was an increase in the contraction force from the Baseline, where both muscles were relaxed, to the CPT, where they were subjected to the cold-painful stimulus. On both muscles, the values decreased during the WarmWater2 and Rest, evidencing that the muscle was able to recover. Regarding the trapezius, the results present very little dispersion between the values during the non-painful epochs. For the triceps, the same is true for the Baseline and Rest epochs.

Concerning the results of the CPT, it is visible that the triceps had, overall, higher RMSA values. Nevertheless, its median value is not only lower than the median of the trapezius, but it is also more similar to the values obtained in WarmWater1 and WarmWater2, seemingly indicating that there was a great dispersion in results among the participants, with half of them not showing a significant reaction in the presence of the cold-painful stimulus, while the opposite was true for the remaining half of the participants. As for the trapezius muscle, a greater dispersion is also observable, especially from the median upwards. There is also observable greater dispersion, especially from the median upwards, and compared with the remaining epochs, presents a higher median.

For the trapezius, there are significant differences between the CPT and all the other epochs, supporting a change in the behavior of the trapezius contraction during the painful stimulus. It is also noticeable differences between the Rest and Warmwater1. The RMSA of the triceps also seems to validate that assumption, as there are significant differences between the CPT and the Baseline and between the CPT and the Rest.

**Figure 11:**
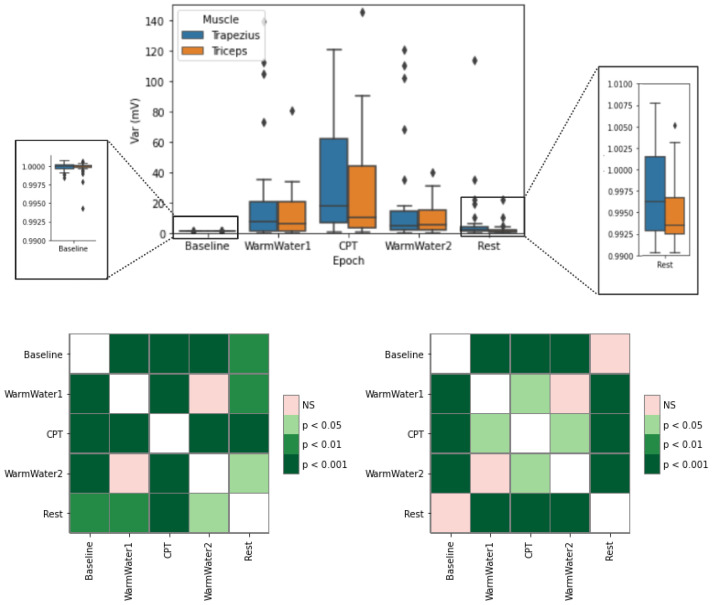
Boxplot of VAR for the trapezius and triceps muscle (**top**) with respective statistical analysis, with *p*-values, with Bonferroni correction, for the trapezius (**bottom left**) and triceps (**bottom right**). The ⋄ stands for outliers.

Figure 11 presents the variance (VAR) results, of both muscles, for the different epochs (the values for the Baseline are close to one due to the normalization). The WarmWater1 shows an almost identical behavior between the two muscles. Even so, the trapezius does appear to have slightly higher values (both with and without considering the outliers) and a median marginally greater value. For the cold water, the data show a reaction to pain, with an increase in the variance. This was especially visible in the trapezius, which had overall considerable higher values, both in the maximum values (excluding outliers) and in the median value (18.06 mV), which is almost double the median of the triceps (9.95 mV). Although there are changes in the variance of the EMG from both muscles, the trapezius showed a more acute reaction to pain than the triceps. After the CPT, there was a decrease, showing a recovery from the pain. The variance is slightly lower relative to the WarmWater1, meaning that, before the beginning of the painful stimulus, the participants applied more power onto their muscles, giving further evidence that participants demonstrated apprehension at the beginning of the CPT. Nonetheless, there are some observed outliers, especially on the trapezius, whose values are closely similar to the ones observed on the CPT. This demonstrates that not all participant’s musculoskeletal systems could recover immediately after the painful stimulus. During the Rest period, the variance roughly returned to values near one, similar to the Baseline, with a slightly higher level of dispersion observed on the trapezius. The statistical analysis for the trapezius shows, as in previous EMG features, very significant differences between the CPT and all the remaining epochs. As for the other epochs, only WarmWater1 and WarmWater2 showed no significant differences from each other. For the triceps, there is a significant difference between the CPT and the other epochs, except for the WarmWater2, which may indicate a slightly longer recovery period of this muscle.

### 4.3. EDA Processing and Analysis

With respect to the EDA signal, the number of SCR (Skin Conductance Response) peaks for each epoch was added up to identify the epoch with larger sympathetic activation. Furthermore, EDA indexes of the sympathetic nervous system (EDASymp) were also calculated based on the findings of [17], who argue that dynamics of the sympathetic component of the EDA signal are represented in the frequency band of 0.045–0.25 Hz.

Table 3 presents a brief description of these features.

**Figure 12:**
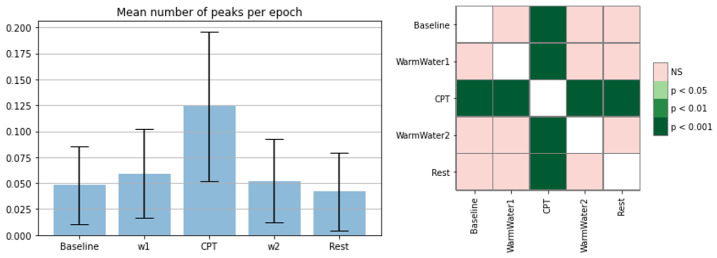
Bar chart of SCR peaks per second with corresponding standard deviation (**left**); *p*-values between different epochs, with Bonferroni correction (**right**).

Figure 12 (left) represents the mean sum of SCR peaks that occurred on a given epoch. Since the epochs varied greatly in length time, the results had to be normalized by time (in seconds). SCR peaks arise from a response to a stimulus. Considering the bar chart and the statistical analysis, it is apparent that the values reached their highest point during the CPT and exhibited statistically significant differences from all the other epochs. These results suggest that the EDA signals of the participants were sensitive to the pain induced by the CPT. As for the remaining epochs, they registered, as expected, fewer peaks, with values pre and post-CPT being relatively similar and with no significant statistical differences between them. The Baseline and WarmWater1, nevertheless, have slightly higher values, which seems to be consistent with what was already hypothesized, regarding the general anticipation of the participants before the CPT.

**Figure 13:**
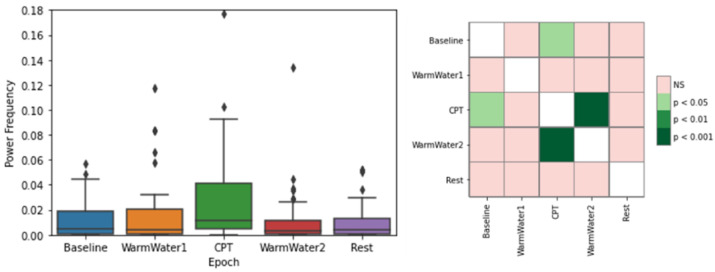
Boxplot of EDASymp values (**left**) and respective *p*-values between different epochs, with Bonferroni correction (**right**). The ⋄ stands for outliers.

The results for the EDASymp are presented in Figure 13. The results show that, overall, although with great levels of dispersion, the epoch with the highest values is the CPT, which seems to support the evidence that the sympathetic outflow increased when the participants were induced into pain.

As for the statistical analysis, it shows that the only significant statistical differences were between the CPT and both Baseline and WarmWater2 epochs. This sustains that the CPT did induce some changes in the sudomotor activity of the participants, in comparison with their initial state.

**Figure 14:**
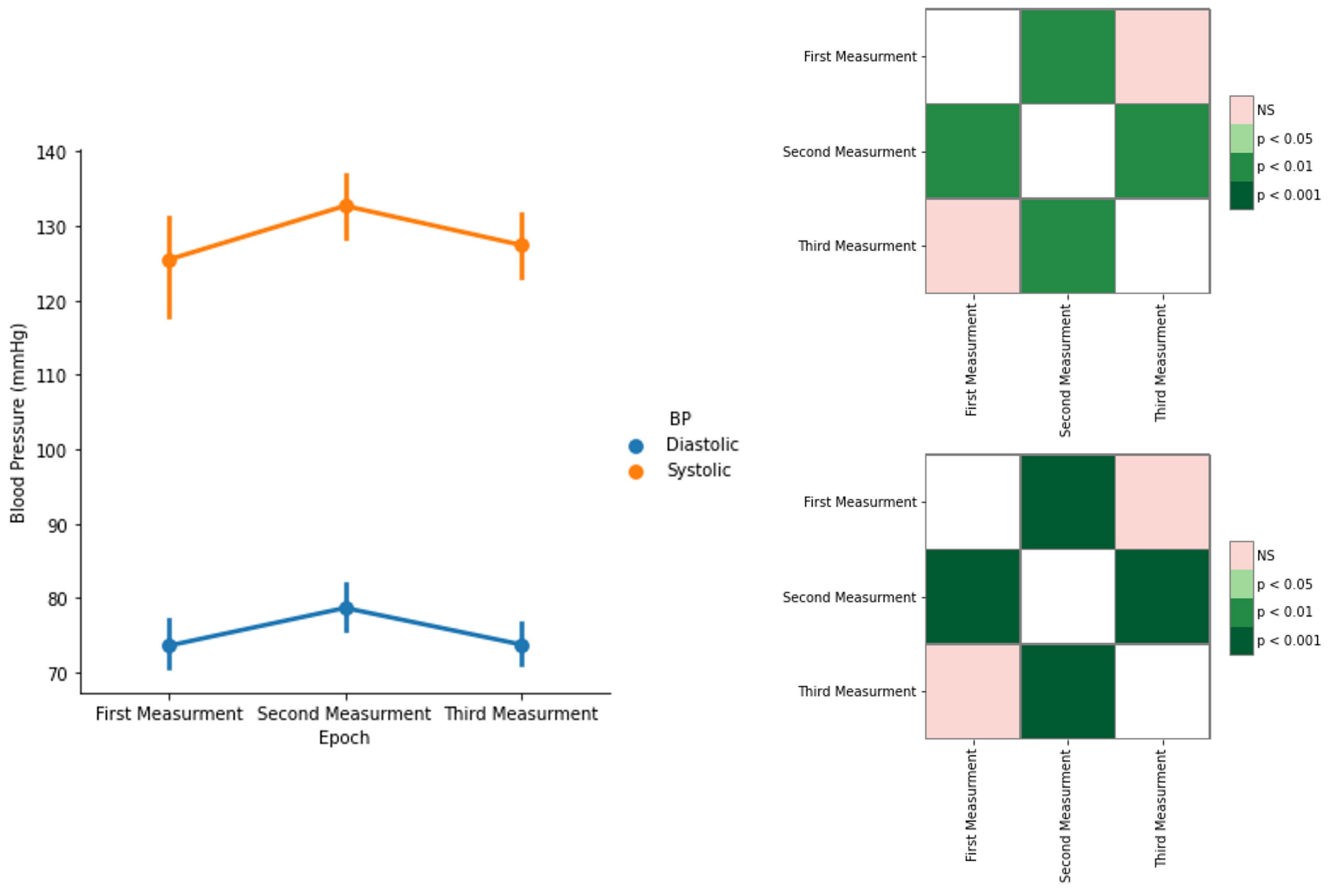
Systolic and diastolic BP values (mean ± level of confidence) (**left**). Statistical differences between the BP measurements and *p*-values, with Bonferroni correction, for systolic (**upper right**) and diastolic (**bottom right**).

Figure 14 represents the evaluation of the systolic and diastolic BP values throughout the study. From the first measurement (before the CPT) to the second measurement (right after the CPT), the systolic and diastolic BP had an increase of 6% and 7%, respectively. In the third BP measurement, five minutes after the ending of the CPT, both systolic and diastolic values returned to their original values. This shows that the participants were able to recover to their initial state, which is supported by the results of the statistical analysis, revealing no significant differences between the first and third BP measurements and significant differences between both the CPT and previous measurements and the CPT and after measurements.

## 5. Discussion

For the study of induced pain, the different physiological systems analyzed seemed to respond in accordance with what was hypothesized. On the ECG, the results on the meanHR were similar to what was described in previous literature [2,3,4,14,18], and is most likely a result of the increased sympathetic outflow on the body. As for the RMSSD, an HRV metric, which is an estimation of the vagally mediated changes, demonstrated a decrease in the parasympathetic outflow to the cardiovascular system during the pain-inducing task. The RMSSD also showed lower values for the two epochs before the CPT, when compared to those that preceded it. This may be a result of a higher level of anxiety felt by the participants before being subjected to pain. The statistical analysis also corroborates this conjecture, as there were no significant differences observed between the Baseline and CPT. The SampEn, which measures the regularity of the signal, demonstrated that the pain induced by the CPT caused a reaction in the participants that lead to a more consistent heartbeat pattern in their cardiac system. The WarmWater2 presented higher values, which implies less predictability and may be attributed to the recovery time that the body needed to return to the initial state by decreasing its HR. Lastly, the mean amplitude of S-peaks showed a progressive decrease in values until the CPT, followed by a progressive increase in the latter epochs. The statistical analysis for this feature also endorsed that there was a response in the S-waves of the ECG to the pain. In general, the results for the ECG features show that the cardiac system seems to react to the cold-painful stimulus. However, it must be pointed out that the RMSSD, the only time-domain HRV metric analyzed, can only contribute to the observation of the PNS, which means that it is not possible to conclude if the reaction observed on the ECG signal is due to the activation of the Sympathetic Nervous System (SNS) or, simply, due to the suppression of the PNS.

The [19] concluded that, when in a state of stress, due to the decrease in parasympathetic activity and increase in sympathetic, the energy will move from the cardiac system into the muscle. An overall analysis of the results for the EMG signal discloses that there was a reaction on part of the musculoskeletal system to the cold pain stimulus. As the RMSA, which is the representation of the non-fatiguing force, both muscles showed greater median values and large dispersion for the CPT epoch—thus corroborating the premise of reaction on the ANS due to the presence of pain, more evident for the trapezius. The statistical analysis shows that the Baseline presents significant differences with all the remaining epochs, indicating that, during the Rest, the muscle did not recover to its original state. The VAR, a representation of the power, also conveys a response of the muscle to the induced pain, showing that the trapezius had a more acute reaction to the pain. For this study, in general, the trapezius seemed to be a consistently more stable source of information in comparison with the triceps. In addition, and according to study [19], our findings indicate an increase in the SNS reactivity, earlier in the procedure, which was further augmented during the CPT intensifying the activity recorded on the EMG.

Overall, the results for the EMG features validate the previous research in the area [15,19]: firstly, the RMSA, where both muscles showed a well-marked of value boxplots for the CPT epoch, thus corroborating the premise of reaction on the ANS due to the presence of pain. The statistical analysis shows that the Baseline presents significant differences with all the remaining epochs, indicating that, during the Rest, the muscle did not recover to the original state, whereas the triceps show differences between the baseline and the remaining epochs, except for the Rest period, indicating that it was able to recover.

For the EDA, it is noticeable that there was a response in the sudomotor activity of the participants when subjected to the painful stimulus. Comparing both time domain and frequency domain analysis, the former yielded better results, especially when taking into account the statistical results. Since the EDA features only measure the SNS, it can be concluded that, indeed, this system was activated during the pain induction task (CPT), in accordance with related works reported [4]. Finally, the BP shows statistically significant differences between consecutive measurements, which is in agreement with the literature [18,20,21].

Overall the results of this study are similar to what was reported in related works [2,3,4,14,15,18,19,20,21]. Nonetheless, this investigation goes further than previous literature, since it uses a higher number of physiological data, and, thus, a deep analysis of the effects of pain in the body.

## 6. Conclusions and Further Research

A new data collection protocol for induced pain is described and evaluated in the present work. For that, four different signals (ECG, EMG, EDA, and BP) were collected. The major innovation in this protocol was the use of a wider range of signals, which allowed for a broader analysis of the ANS reactivity on the various body systems.

Under this study, a deep evaluation of physiological data was performed, and, thus, a more concrete analysis of the effects of pain in the body was provided.

From the ECG, a significant increase in the HR and a decrease in the PNS activity were observed, based on the HRV metrics calculated, as a result of the cold-painful stimulus. Furthermore, the ECG also suffered a change in its amplitude, which was particularly noticeable in the S-wave evaluation.

The EMG, recorded both on the trapezius and triceps muscles, also showed changes brought on by the pain-inducing protocol—mainly an increase in amplitude during the CPT, in comparison with the other resting periods. Moreover, the results on the trapezius muscles seemed to indicate that the stabilization of the values after an initial increase was crucial to withstanding the painful stimulus for the participants who completed the CPT.

Both time and frequency domain features on the EDA demonstrated an increase in the values during the CPT and hence an increase in the SNS.

Finally, the BP shows statistically significant differences between consecutive measurements, which is in accordance with the literature.

Since this study uses a variety of physiological signals, future work should be concerned with the study of the signals interrelation in the process of pain and devoted to multimodal classification providing further reliable measurements of pain. Moreover, a setback in this study is the short length of time recordings of the CTP, which hindered the study of the majority of HRV metrics. Thus, a protocol with an increased length of the CPT would allow the investigation into the influence of the SNS on the cardiovascular system.

## Figures and Tables

**Table 1:** Description of the extracted ECG features.

Feature	Description
Mean HR	Number of beats per minute (mean)
R peaks	Maximum value of the ECG cycles (upward deflections)
S peaks	Minimum value of the ECG cycles (downward deflections)
RMSSD	Root Mean Square of Successive Differences between normal heartbeats. It is a reflection of the beat-to-beat difference in the HR and is used to estimate the alteration of the HRV caused by the vagus nerve.
pNN50	Percentage of successive RR intervals that are greater than 50 ms, associated with the Parasympathetic Nervous System (PNS) activity
SampEn	Measures the regularity and complexity of a given signal. Smaller values indicate a regular and predictable signal

**Table 2:** Description of the extracted EMG features.

Features	Description
RMSE and RMSA	Root Mean Square for Electromyogram and Amplitude, respectively. Related to the constant force and not-fatiguing contractions of the muscles.
VAR	Variance. It allows for expressing the power of the EMG signal.

**Table 3:** Description of the extracted EDA features.

Features	Description
SCR peaks	The number of SCR peaks (cumulative calculation of SCR peaks for each participant and epoch averaged and normalized by time in seconds).
EDASymp	Indexes of the sympathetic nervous system for the frequency band of 0.045–0.25 Hz [17].

## Data Availability

The data are protected by the GDPR and cannot be publicly available.
